# Using the Conceptual Framework for Examining Sport Teams to Understand Group Dynamics in Professional Soccer

**DOI:** 10.3390/ijerph192315782

**Published:** 2022-11-27

**Authors:** Inmaculada González-Ponce, Jesús Díaz-García, José C. Ponce-Bordón, Ruth Jiménez-Castuera, Miguel A. López-Gajardo

**Affiliations:** 1 Department of Psychology and Anthropology, University of Extremadura, 06006 Badajoz, Spain; 2 Department of Didactics of Musical, Plastic and Corporal Expression, University of Extremadura, 10003 Cáceres, Spain

**Keywords:** execution, group dynamics, resilience, team performance, team sports

## Abstract

(1) Background: The aim of this study is to propose a model of the interactions of group dynamics using the conceptual framework to examine sports teams; (2) Methods: The hypothesized model includes measures of group structure (authentic leadership, perceived justice, coaching competency, role clarity/ambiguity, and role conflict), group cohesion (cohesion and team conflict), and group processes (collective efficacy and transactive memory systems). Participants were 581 professional soccer players (*M* = 24.51, *SD* = 3.73; 356 males and 225 females) who completed a multisection questionnaire assessing group dynamics variables; (3) Results: The results show that coach leadership predicts coaching competency and perceived justice, and both competency and justice predict role ambiguity and role conflict. Furthermore, role ambiguity and role conflict predict group cohesion and team conflict, whereas group cohesion and team conflict both predict the transactive memory system. Finally, collective efficacy is predicted by the transactive memory system; (4) Conclusions: The results suggest the importance of coach behavior (leadership, justice, and competency) and group processes to improving team functioning in a professional sports context.

## 1. Introduction

Recent research on team dynamics highlights the importance of group processes to achieve good team functioning [1,2,3]. Many approaches and variables have been studied to determine what occurs in sports teams in order to optimize performance. In this regard, important relationships have been found among team variables involving coaches and players, which have shown a strong connection with successful performance [4,5,6].

**Figure 1:**
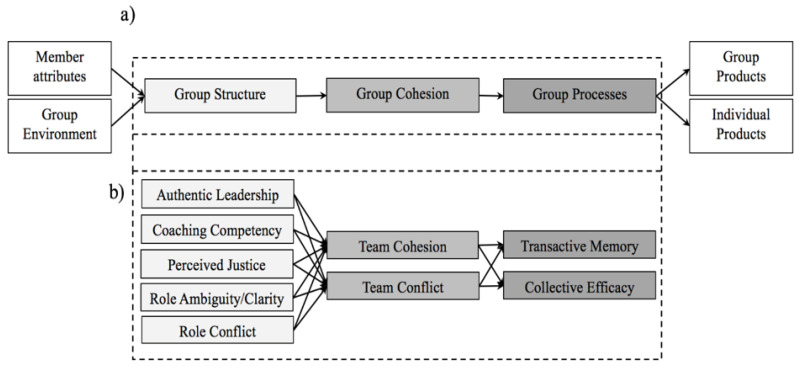
(**a**) A conceptual framework for the study of sports teams developed by Carron and Eys [7]. (**b**) Proposed model of team dynamics in sports.

Due to the concerns about the relationships among the variables that influence sports teams’ performance, Carron and Eys [7] proposed a conceptual framework to study sports teams, with three large, sequentially related blocks: inputs, throughputs, and outputs. Inputs are the characteristics of the group’s environment and the attributes of individual group members. Throughputs are: (a) group structure (group position, group status, group roles, group norms, and group leadership); (b) group cohesion; and (c) group processes (team goals, group cooperation, and competition, attribution in sport, group communication, and collective efficacy). Lastly, outputs, the major consequences of groups, are individual outcomes and team outcomes (see Figure 1a).

The reviewed literature on the importance of team dynamics with regard to optimizing sports teams’ performance used an analytic approach, where a number of variables were examined. These investigations analyzed the relations between the different blocks, centered mainly on how some throughput variables (group structure, group cohesion, and group processes) are related to each other [5,8,9,10]. However, Carron and Eys [7] pointed out that studies that include the largest possible number of variables that affect group processes are needed in order to achieve a more global vision of the interactions that take place in sports teams. To our knowledge, there are no studies that propose a model to evaluate the relations between the three throughput components. Thus, this paper will try to determine the relationships between these components (group structure, group cohesion, and group processes) in order to better understand group dynamics within sports teams (see Figure 1b).

As mentioned, the model proposed by Carron and Eys [7] divided throughputs into three blocks: group structure, group cohesion, and group processes. Within group structure, it has been shown that the establishment of clear norms and roles, a small group size, and appropriate coaching behaviors are related to improvements in team sports [3,10,11,12,13].

According to the model of Carron and Eys [7], previous research has verified the coach behaviors of importance within a group structure as a guide to creating good group cohesion [3,12,14,15]. Many theories have been used to explain the coach’s importance in sports [2,16], but we specifically analyze authentic leadership, perceived justice, and coaching competency in this study, as these variables have gained importance to explain team dynamics in the sports setting [6,14].

Authentic leadership (i.e., “a pattern of leader behavior …of greater self-awareness, an internalized moral perspective, balanced processing of information, and relational transparency on the part of leaders working with followers, fostering positive self-development”; [16], p. 94) illustrates a simpler, more transparent, and reliable leadership [17]. A leader’s authentic behavior can achieve benefits for the team, not only regarding the group’s well-being but also its functioning [18,19]. Perceived justice (athletes’ perceptions of fair treatment received from organizations; [20]) can also be a relevant variable as, in sports teams, not all players play the same or are equally important. Thus, if players perceive their coach’s fair decision-making, this can produce positive behaviors among teammates and prevent conflicts within the team [14]. Furthermore, generating a positive perception of coaching competency (athletes’ perceptions of their head coach´s ability to affect their learning and performance; [21]) in the players through the transmission of motivation, technical corrections, or correct strategies can help the group to evolve and develop positively [6]. Summing up, when the coach can lead effectively, treating the players fairly and showing good skills, then team performance may improve. In fact, investigations have revealed positive relationships between authentic leadership, perceived justice, and coaching competency and cohesion in sports teams [12].

On another hand, roles have also shown a strong link with team functioning. Specifically, role ambiguity (defined as a lack of clear information associated with one’s role; [22]) and role conflict (defined as the presence of incongruent expectations about an incumbent role; [22]) have been associated with positive (e.g., group cohesion) and negative consequences (tension, dissatisfaction, burnout, or less commitment; [23]). In the sports setting, Carron and Eys [24] found that players who felt unclear about their role responsibilities perceived their team to be less integrated in terms of its approach to team play, and they reported lower levels of attraction to the team. Thus, when players feel unclear about their functions, and they receive contradictory information about what they must do, this causes a lack of coordination among them and lowers their cohesion when resolving situations during the competition.

Therefore, coach behaviors (leadership, perceived justice, and coaching competency) and roles (role ambiguity and role conflict) within a group structure clearly predict group cohesion. Such group cohesion is the central factor of Carron and Eys´s model [7]. This construct has been defined as “a dynamic process that is reflected in the tendency for a group to stick together and remain united in the pursuit of its instrumental objectives and/or for the satisfaction of member affective needs” [25], and it has been one of the most studied factors that leads to an optimal functioning of the group [5,26,27,28]. Along with group cohesion, the latest research has incorporated team conflict (defined as “a process in which one party perceives that its interests are being opposed or negatively affected by another party”; [29], p. 517), a concept which can be considered as opposed to cohesion. Although the original model of Carron and Eys [7] did not include team conflict, this construct reflects maladaptive behaviors that may arise in the group [30] and could provide relevant information about team dynamics [31]. Therefore, the examination of how both cohesion and conflict behave conjointly can contribute more knowledge about the relations among players [31,32].

Accordingly, group cohesion or team conflict can both have an impact on group processes, the third block of throughputs within Carron and Eys´s model [5,9,33,34]. Within this block are variables of a different nature, such as team goals, group communication, or collective efficacy. However, this research focuses on collective efficacy and the transactive memory system (TMS), as they are variables that reflect a group’s possible performance [35].

In this sense, both collective efficacy (defined by Zaccaro and colleagues as “a sense of collective competence shared among individuals when allocating, coordinating, and integrating their resources in a successful, concerted response to specific situational demands” [36], p. 309) and TMS have been the variables whose relationship with cohesion have been contrasted [33,37,38,39,40]. TMS is a more global concept than communication and cooperation, referring to one of the most relevant theories of mental processes in teams, which specifically mentions a cooperative division of work to learn, remember, and communicate relevant team knowledge [10,38,39]. Some of the research findings have revealed that cohesion among teammates may help to improve collective knowledge [5,41].

After analyzing the context of group dynamics, our present study extends previous studies by creating a model that explains the interactions among some of the variables that influence team dynamics [7]. 

Thus, the aim of this study is to propose an explanatory model of group dynamics in professional soccer using the conceptual framework to examine team sports [7]. The model establishes a linear relationship between group structure, group cohesion, and group processes. For this purpose, several variables were used to measure the components of group structure (authentic leadership, coaching competency, perceived justice, role clarity/ambiguity, and role conflict), group cohesion (group cohesion and team conflict), and group processes (collective efficacy and TMS). On the basis of this aim and in general terms, we hypothesized that the proposed model adequately fits the data, that group structure predicts group cohesion, and that this would, in turn, predict group processes. However, considering the large number of possible relationships between all variables (see the results section for the four different models tested), we did not formulate specific hypotheses because, to our knowledge, there are no studies that have analyzed the relations of the target variables of the current study in the same project. 

## 2. Materials and Methods

### 2.1. Participants

The participants were 581 professional soccer players, aged between 15 and 39 years (*M* = 24.51, *SD* = 3.73) and with a mean soccer experience of 14.01 years (*SD* = 5.16), who participated in the Spanish Soccer League. Regarding gender, there were 356 males from 18 teams who participated in the third-highest division and 225 females from 13 teams who participated in the highest division.

### 2.2. Instruments

#### 2.2.1. Authentic Leadership

We used an adaptation for sports of the Authentic Leadership Questionnaire (ALQ: [16]). This questionnaire consists of 12 items and 4 factors: relational transparency (3 items, e.g., “says exactly what he or she means”), internalized moral perspective (3 items, e.g., “demonstrates beliefs that are consistent with actions”), balanced processing (3 items, e.g., “listens carefully to different points of view before coming to conclusions”), and self-awareness (3 items, e.g., “seeks feedback to improve interactions with others”). Players responded to all items on a seven-point scale ranging from *never* (1) to *always* (7). A confirmatory factor analysis (CFA) shows an acceptable model fit: chi-square divided by degrees of freedom (*χ*^2^/*df*) = 4.58, the Comparative Fit Index (CFI) = 0.91, the Tucker–Lewis index (TLI) = 0.90, the Root-Mean-Square Error of Approximation (RMSEA) = 0.08, and Standardized Root-Mean-Square Residual (SRMR) = 0.05.

#### 2.2.2. Coaching Competency

The Spanish version [42] of the Athletes’ Perceptions of Coaching Competency Scale II–High School Teams (APCCS II-HST: [43]) was used. This instrument begins with the introductory item “how competent is your head coach in his/her ability to…”, and it has a total of 15 items divided into 4 factors: motivation competency (4 items, e.g., “…help players to maintain confidence in their ability to perform when they are performing poorly”), game-strategy competency (4 items, e.g., “…make effective strategic decisions in pressure situation during competition”), technique competency (4 items, e.g., “…detect subtle technical errors by players during practices”), and character-building competency (3 items, e.g., “…effectively promote good sportsmanship in players”). Players responded to all items on a five-point scale ranging from *complete incompetence* (1) to *complete competence* (5). The CFA shows an acceptable model fit (*χ*^2^/*df* = 4.51, CFI = 0.98, TLI = 0.98, RMSEA = 0.08, SRMR = 0.04).

#### 2.2.3. Perceived Justice

To assess perceived justice, we relied on Colquitt´s research [44], using a 12-item scale with 4 factors: procedural justice (3 items, e.g., “my coach is consequential and replaces a player when he/she is underperforming”), distributive justice (3 items, e.g., “my coach rewards with enough playing time, taking into account the contribution to the team”), interpersonal justice (3 items, e.g., “My coach treats the players with respect in training and matches”), and informational justice (three items, e.g., “my coach explains and discusses his or her tactical decisions”). Players responded to all items on a seven-point scale ranging from *never* (1) to *always* (7). The CFA shows an acceptable model fit (*χ*^2^/*df* = 4.17, CFI = 0.93, TLI = 0.91, RMSEA = 0.07, SRMR = 0.04).

#### 2.2.4. Role Clarity/Ambiguity

The Spanish version [45] of the Role Ambiguity Scale (RAS: [46]) was used. This instrument has 12 items divided into 3 factors: task (6 items, e.g., “I understand all of my responsibilities”), evaluation ambiguity (3 items, e.g., “It is clear to me how my role responsibilities are evaluated”), and role-consequences ambiguity (3 items, e.g., “It is clear to me what will happen if I fail to carry out my role responsibilities”). Players responded to all items on a nine-point scale ranging from *strongly disagree* (1) to *strongly agree* (9). Higher ratings of agreement indicate greater role clarity and, hence, less role ambiguity. The CFA shows an acceptable model fit (*χ*^2^/*df* = 3.08, CFI = 0.95, TLI = 0.94, RMSEA = 0.06, SRMR = 0.03).

#### 2.2.5. Role Conflict

To assess role conflict, we used a six-item scale employed by Leo et al. [9] adapted from the instrument developed by Beauchamp and Bray [47]. An example of an item of the role-conflict scale is: “I am expected to play in a way that is inconsistent with the way I would rather play”. Responses were rated on a five-point scale ranging from *strongly disagree* (1) to *strongly agree* (5). Thus, higher ratings of agreement indicate greater role conflict. The CFA shows an acceptable model fit (*χ*^2^/*df* = 2.21, CFI = 0.98, TLI = 0.97, RMSEA = 0.04, SRMR = 0.03).

#### 2.2.6. Group Cohesion

The Spanish version [48] of the Group Environment Questionnaire (GEQ: 1985) was used. This scale has 12 items divided into 4 factors: group integration-social (3 items, e.g., “the members of our team like to get together in situations other than trainings and matches”), group integration-task (3 items, e.g. “the team members unite their efforts to achieve the goals during trainings and matches”), individual attraction to group-social (3 items, e.g., “If there is any problem during the training sessions, all the players get together to overcome it”), and individual attraction to group-task (3 items, e.g., “I like the way this team plays”). Players responded to all items on a nine-point scale ranging from *strongly disagree* (1) to *strongly agree* (9). The CFA shows an acceptable model fit (*χ*^2^/*df* = 2.71, CFI = 0.94, TLI = 0.92, RMSEA = 0.06, SRMR = 0.04).

#### 2.2.7. Team Conflict

A six-item scale adapted from the instrument developed by Jehn [49] and adapted by Tekleab et al. [31] was used. This instrument begins with the introductory item “how frequently…”, and it has a total of 6 items divided into in 2 factors: task conflict (3 items, e.g., “…were there differences of opinion on your team?”) and relationship conflict (3 items, e.g., “…was there tension among members on your team?). Players responded to all items on a seven-point scale ranging from *never* (1) to *always* (7). The CFA shows an acceptable model fit (*χ*^2^/*df* = 2.05, CFI = 0.99, TLI = 0.98, RMSEA = 0.06, SRMR = 0.02).

#### 2.2.8. Transactive Memory System

The Spanish version [50] of TMS was used [51]. The instrument used has a total of 15 items divided into 3 factors: specialization (5 items, e.g., “Different team members are responsible for expertise in different areas”), credibility (5 items, e.g., “I am comfortable accepting procedural suggestions from other team members”), and coordination (5 items, e.g., “Our team has very few misunderstandings about what to do”). Players responded to all items on a five-point scale ranging from *strongly disagree* (1) to *strongly agree* (5). The CFA shows an acceptable model fit (*χ*^2^/*df* = 2.77, CFI = 0.96, TLI = 0.95, RMSEA = 0.05, SRMR = 0.03).

#### 2.2.9. Collective Efficacy

To assess collective efficacy, we used a scale developed by Leo et al. [52]. The instrument has a total of six items and a single factor (e.g., “how do you rate the team in the attack phase?”). Players responded to the items on a five-point scale ranging from *bad* (1) to *excellent* (5). The CFA shows an acceptable model fit (*χ*^2^/*df* = 2.76, CFI = 0.97, TLI = 0.95, RMSEA = 0.06, SRMR = 0.04).

### 2.3. Procedure

The study received ethical approval from the University of Extremadura. All participants were treated according to the American Psychological Association’s ethical guidelines regarding consent, confidentiality, and anonymity of responses. Moreover, to carry out data collection, we contacted the clubs and coaches of different teams of Second Division B and First Division to request the inclusion of their teams in the research. If they were willing to participate in the research, we informed them about the objectives and procedures to be carried out. Later, players were informed about the research objectives and assured that their participation was voluntary and that their responses would be treated confidentially. Participants completed questionnaires in the locker room without the presence of the coach, individually, and in a suitable climate. The process took approximately 30 min. All participants included in the present study are Spanish speakers. However, the principal investigator was present while the players completed questionnaires in order to resolve any doubts that might arise during the process.

### 2.4. Data Analysis

Initially, descriptive statistics and internal consistency reliability estimates for all study variables were calculated using SPSS 21.0 software. At this time, a CFA was also conducted on the study questionnaires to test the psychometric properties using Mplus 7.3 [53]. We subsequently performed structural equation modeling (SEM) with maximum likelihood estimation (MLE) in order to determine the predictive capacity of the study variables. Leadership, coaching competency, perceived justice, role ambiguity, TMS, team conflict, and group cohesion were estimated as global factors using the specific factors (mean scores) as observable indicators. For collective efficacy and role conflict, we created two separate parcels composed of three randomly selected items because of the reduced number of items that formed the factors.

In this study, we used the following fit indexes: *χ*^2^, *df*, CFI, TLI, RMSEA, and SRMR. According to Schumacker and Lomax [54], the incremental indexes (CFI and TLI) indicate an acceptable fit when they obtain values of 0.90 or higher. Regarding RMSEA and SRMR, 0.08 has been established as an acceptable cut-off point [55]. We also used three information criteria indices: the Akaike information criterion (AIC), the Bayesian information criterion (BIC), and the sample size adjusted BIC (ABIC). Finally, to test the indirect effects, we used bootstrapping resampling procedures (*N* = 10,000) to compute 95% bias-corrected confidence intervals (95% BcCI). This model was estimated using the maximum likelihood (ML) estimator (MLE as bootstrapping is not yet available in Mplus).

## 3. Results

### 3.1. Descriptive Statistics and Cronbach’s Alpha Coefficients

Means, standard deviations, normality, and Cronbach’s alpha coefficients for each variable are presented in Table 1. Regarding means, in general, participants obtained scores above the midpoint of the scale for authentic leadership, coaching competency, perceived justice, role ambiguity, cohesion, TMS, and collective efficacy. Participants also obtained scores for role conflict and team conflict below the midpoint of the scales.

### 3.2. Model Testing

**Figure 2:**
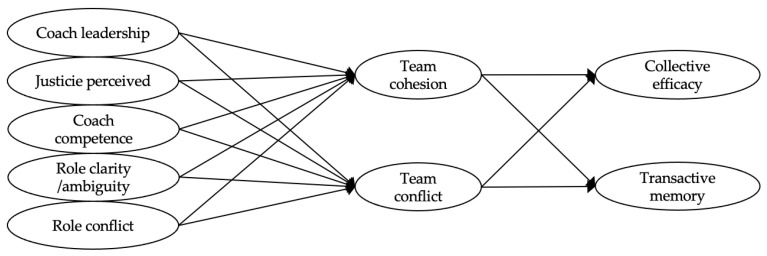
The hypothesized Model 1 of the relationships among variables.

Drawing on the model of Carron and Eys [7] (see Figure 1), the aim of this study is to test Model 1 (Figure 2), in which group structure variables (authentic leadership, coaching competency, perceived justice, role clarity/ambiguity, and role conflict) predict group cohesion (group cohesion and team conflict), and group cohesion predicts group processes (collective efficacy and TMS). The results from structural equation modeling (see Table 2) indicate that the model does not adequately fit the data (CFI = 0.809, TLI = 0.784, RMSEA = 0.091, SRMR = 0.250), so the model was respecified.

**Figure 3:**
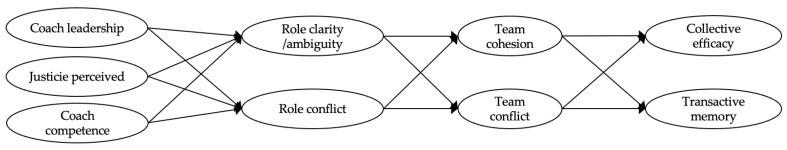
The hypothesized Model 2 of the relationships among variables.

Based on previous research [9,32,56], within the group structure, the coach’s behaviors (authentic leadership, coaching competency, and perceived justice) are presented as antecedents of the roles—role clarity/ambiguity and role conflict—and the roles as antecedents of group cohesion (Model 2; see Figure 3). The fit of this model (see Table 2) is slightly better, but still inadequate (CFI = 0.817, TLI = 0.793, RMSEA = 0.089, SRMR = 0.220).

**Figure 4:**
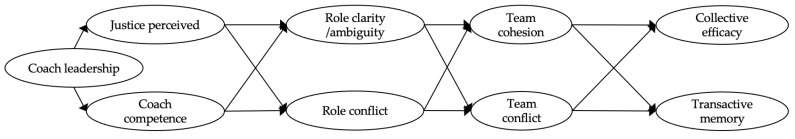
The hypothesized Model 3 of the relationships among variables.

For the next step, following the findings of recent research [2,57,58], we included authentic leadership as an antecedent of coaching competency and perceived justice (Figure 4). The results from structural equation modeling (see Table 2) indicate an acceptable fit to the data (CFI = 0.914, TLI = 0.903, RMSEA = 0.061, SRMR = 0.057).

**Figure 5:**
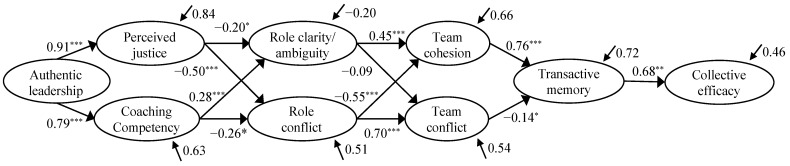
Structural equation model of the relationships among variables. * *p* < 0.05, ** *p* < 0.01, *** *p* < 0.001.

Based on the research developed by Filho et al. [5] and Leo et al. [32], we proposed Model 4 (Figure 5), in which group structure and group cohesion are the same as in Model 3, but TMS was included as an antecedent of collective efficacy. The fit of this model (see Table 2) is acceptable (CFI = 0.918, TLI = 0.908, RMSEA = 0.059, SRMR = 0.060), and it is considered the best model.

Regarding the regression coefficients of the selected model (Model 4), the first level of the model establishes authentic leadership as a strong positive predictor of perceived justice (*β* = 0.91, *p* < 0.001) and coaching competency (*β* = 0.79, *p* < 0.001). At the second level, perceived justice and coaching competency positively predict role clarity (*β* = 0.20, *p* = 0.023 and *β* = 0.28, *p* < 0.001, respectively) and negatively predict role conflict (*β* = −0.50, *p* < 0.001 and β = −0.26, *p* = 0.021, respectively). Subsequently, role clarity positively predicts group cohesion (*β* = 0.45, *p* < 0.001) and negatively predicts team conflict (*β* = −0.09, *p* = 0.130). The same outcome trend was observed for role conflict but with the opposite sign, that is, role conflict negatively predicts group cohesion (*β* = −0.56, *p* < 0.001) and positively predicts team conflict (*β* = 0.70, *p* < 0.001). At the fifth level of the model, TMS is positively predicted by group cohesion (*β* = 0.76, *p* < 0.001) and negatively predicted by team conflict (*β* = −0.14, *p* = 0.049). Finally, at the last level of the model, collective efficacy is positively predicted by TMS (*β* = 0.68, *p* < 0.001).

### 3.3. Indirect Effects

The total indirect effects are shown in Table 3. In this section, to simplify the results, only the significant indirect effects are explained. Firstly, significant indirect effects were found from leadership to role clarity through justice (*β* = 0.18, SE = 0.080, *p* = 0.024, BC 95% CI = 0.049, 0.311) and competence (*β* = 0.22, SE = 0.065, *p* < 0.001, BC 95% CI = 0.114, 0.329); and to role conflict through justice (*β* = −0.46, SE = 0.087, *p* < 0.001, BC 95% CI = −0.603, −0.317) and competence (*β* = −0.21, SE = 0.099, *p* = 0.036, BC 95% CI = −0.371, −0.045).

Secondly, the results revealed significant indirect effects from perceived justice to cohesion through role clarity (*β* = 0.09, SE = 0.044, *p* = 0.047, BC 95% CI = 0.015, 0.161) and through role conflict (*β* = 0.28, SE = 0.062, *p* < 0.001, BC 95% CI = 0.179, 0.381); and to team conflict through role conflict (*β* = −0.35, SE = 0.068, *p* < 0.001, BC 95% CI = −0.465, −0.243). Furthermore, significant indirect effects were observed from coaching competency to cohesion through role clarity (*β* = 0.12, SE = 0.042, *p* = 0.003, BC 95% CI = 0.057, 0.194); and to team conflict through role conflict (*β* = −0.18, SE = 0.092, *p* = 0.045, BC 95% CI = −0.336, −0.033). Finally, group cohesion showed a significant indirect effect to collective efficacy through TMS (*β* = 0.52, SE = 0.048, *p* < 0.001, BC 95% CI = 0.438, 0.595).

## 4. Discussion

The aim of this study is to propose a model to understand group dynamics in professional soccer using the conceptual framework to examine sports teams proposed by Carron and Eys [7]. Following the hypothesized model, and after checking different models based on previous research [2,9,32,56,57,58], the results indicate that the proposed Model 4 developed to explain team dynamics obtains adequate fit indexes and helps to explain the group behaviors that occur in a sports environment, supporting the general hypothesis noted above. Thus, we were able to test a model that verifies the relations established between group structure, group cohesion, and group processes.

The results of this study support the relationship between the variables shown in the model of Carron and Eys [7]. Moreover, the results also expand their theoretical model as follows: (a) The original model proposed by Carron and Eys [7] emphasizes that different antecedents, such as coach leadership and roles, are related to cohesion. The present study corroborates this and adds the fact that other coach behaviors (perceived justice and coaching competency) are also a part of the group structure in a sports team.

In addition, the results establish relationships between coaching behavior and roles, which was not an objective of the original model, but these results add new knowledge to the relationships among the group structure variables as antecedents of cohesion. (b) The model of Carron and Eys [7] proposes group cohesion as a mediator between group structure and group processes. However, in the group-cohesion block, only cohesion appears as a positive variable in this study, and team conflict is introduced at the same level as cohesion as a mediator between group structure and group processes. (c) Furthermore, another aspect to underline is the analysis of the relations between group processes, including TMS as an antecedent of collective efficacy. (d) Finally, as proposed by Paradis and Loughead [37], indirect effects must be present to determine the relationships between antecedents and consequences. This study adds indirect effects as a fundamental aspect for future research.

Examining in more depth the relationship between the variables of Model 4, first, we note that authentic leadership positively and significantly predicts perceived justice and coaching competency. Traditionally, studies have examined different coach behaviors and their relationship with variables such as satisfaction, cohesion, and performance [6,12,18], but few works have considered the connection among such coach behaviors. To our knowledge, only four previous studies have found similar results to those of our study, where leadership is an antecedent of coaching competency and perceived justice [2,57,58,59]. Thus, we can corroborate that players who perceive authentic leadership rate their coaches as more competent and fairer; that is, they perceive that their coach motivates them more, gives adequate instructions in training sessions and games, performs appropriate substitutions, and correctly distributes the game minutes, aspects that are fundamental to maintain an adequate training climate.

Secondly, we can observe in Figure 2 that coaches who are perceived by their players as competent and fair in their decisions are associated with players’ greater role clarity and fewer role conflicts. Similar results were found by Bosselut et al. [8], where a lower perception of the coach’s technical and tactical instruction (coaching competency) is linked to a strong perception of role ambiguity. Likewise, high levels of role ambiguity and role conflict are related to high levels of tension, a decrease in commitment, and low satisfaction [60,61]. Therefore, it is essential for coaches to have high competency and to be fair in their decisions, as this will help the players feel clearer about their functions on the team and will decrease role conflict about their tasks in the sports setting. When, through their instructions, decisions, and actions, coaches adequately transmit to the players their function on the team and how to perform it, there will be fewer role conflicts. In this respect, the linear relations between authentic leadership and perceived justice and coaching competency and these latter variables with role clarity and role conflict are also justified by the indirect effects, where we observe the mediation of perceived justice and coaching competency between leadership and roles. Therefore, we verify the existence of different sequential levels within the group structure.

Thirdly, we can observe in the model how role clarity positively predicts cohesion and negatively predicts team conflict. In other words, high role ambiguity leads to lower group cohesion and higher team conflict. Similar results were found by Eys and Carron [24] and Johnson [62], revealing a strong relationship between role ambiguity and group cohesion. Moreover, it can also be seen that higher role conflict is related to higher levels of team conflict and lower group cohesion. Hence, if coaches clarify the players’ functions, and the players do not receive contradictory information about their roles, the relationships among the teammates will improve, and team conflicts may decrease.

Fourthly, it can be observed in the model that group cohesion significantly and positively predicts TMS, and team conflict negatively predicts TMS. Similar results were found by Leo et al. [32] in a sample of female soccer players, in which the authors reported that the players who felt more attracted to and integrated within the group regarding task aspects had higher levels of TMS. Therefore, teams that have lower team conflict and higher cohesion can develop a higher TMS; that is, better knowledge of the functions performed by each player in the team, greater trust in teammates’ capacities, and higher coordination in the team’s activity.

Finally, at the last level of the model, collective efficacy is predicted by TMS [5,32]. In this sense, the results seem to corroborate that players who perceive a higher TMS in their sports teams will associate it with a higher collective efficacy. This indicates that, if each team member is specialized in a sports sphere, believes in what the other teammates are doing, and the team members coordinate to develop their respective functions, the perception of collective efficacy within the sports setting will increase. In this respect, the linear relation between cohesion, TMS, and collective efficacy is confirmed by the indirect effects, which indicate that TMS is a mediator between cohesion and collective efficacy.

### Limitations, Future Perspectives, and Conclusions

One limitation of our work is the correlational nature of the study, which does not facilitate causal inferences. Although the hypothesized relations are based on a solid theoretical framework and research studies, intervention studies are needed to ensure the relations among the variables. Secondly, another limitation is that it is a cross-sectional study, where the variables are measured at a single moment in the season. Taking into account the dynamic nature of the variables, in order to provide additional indications of the direction of the relations, longitudinal studies are needed to observe how the variables fluctuate over time. Thirdly, the sample of the study only comprised soccer teams, which limits the generalization of our results to other team sports. Although group dynamics are similar in different team sports, the number of players and the characteristics of the sport can influence the behaviors of the coach and the players. Therefore, it would be interesting for future studies to consider other team sports to increase the external validity of the results.

Besides the abovementioned future perspectives, it would be interesting to include the inputs, namely, the characteristics of the group environment (e.g., group size), the attributes of individual group members (e.g., the nature of the group composition), and the outputs or major consequences in groups: individual outcomes and team outcomes (e.g., team performance). In addition, variables included in the emerging throughputs, such as team resilience or athlete leadership, can be added.

## 5. Conclusions

Despite the limitations, we consider that this work makes a unique contribution to the literature because it has tested a model of group dynamics based on the previously presented models [5,7,32], including the interrelationships between numerous variables that have a great impact on group dynamics. Knowledge of this model is essential for coaches and sports psychologists because it shows them how important coach behavior is for a series of consequences that influence players’ behavior and how the players, in turn, affect each other to optimize the functioning of a sports team.

Taking into account the relationships found, the findings show the importance of coach behavior for the sports team, because the players’ perception of the coach’s leadership style, fair decisions, and competency determines either higher role clarity or role conflict in the sports setting and, as a consequence, the existence of cohesion or, contrariwise, conflict in the sports team. Lastly, the abovementioned antecedents influence the sports team’s TMS and perception of collective efficacy, concepts that are highly related to sports performance.

## Figures and Tables

**Table 1:** Descriptive statistics, normality, and internal consistency.

	M	SD	Skewness	Kurtosis	α
Authentic leadership	5.39	0.86	−0.70	0.70	0.87
Coaching competency	4.07	0.53	−0.47	−0.33	0.90
Perceived justice	5.63	0.80	−0.95	0.96	0.87
Role clarity/role ambiguity	7.65	1.33	−1.30	1.91	0.95
Role conflict	1.67	0.62	1.08	1.01	0.73
Group cohesion	7.26	1.03	−0.79	0.78	0.82
Team conflict	2.10	0.91	1.07	1.18	0.84
Transactive memory system	4.15	0.49	−0.35	−0.19	0.84
Collective efficacy	3.91	0.51	−0.15	0.20	0.77

**Table 2:** Fit Indices of the different models.

	X^2^	df	CFI	TLI	RMSEA	SRMR	AIC	BIC	ABIC
Model 1	1789.699	310	0.809	0.784	0.091	0.250	32,074.2888	32,488.940	32,187.351
Model 2	1725.402	310	0.817	0.793	0.089	0.220	31,987.737	32,402.388	32,100.799
Model 3	975.045	310	0.914	0.903	0.061	0.057	31,127.015	31,541.667	31,240.078
Model 4 (final)	945.994	311	0.918	0.908	0.059	0.060	31,092.083	31,502.369	31,203.955

**Table 3:** Indirect effects of the different models.

Parameter	β
Authentic leadership→Role clarity	0.402 ***
Authentic leadership→Role conflict	−0.668 ***
Authentic leadership→Cohesion	0.551 ***
Authentic leadership→Team conflict	−0.507 ***
Authentic leadership→Transactive memory system	0.491 ***
Authentic leadership→Collective efficacy	0.333 ***
Perceived justice→Cohesion	0.368 ***
Perceived justice→Team conflict	−0.373 ***
Perceived justice→Transactive memory system	0.333 ***
Perceived justice→Collective efficacy	0.226 ***
Coaching competency→Cohesion	0.271 ***
Coaching competency→Team conflict	−0.211 **
Coaching competency→Transactive memory	0.236 **
Coaching competency→Collective efficacy	0.160 **
Role clarity→Transactive memory system	0.354 ***
Role clarity→Collective efficacy	0.240 ***
Role conflict→Transactive memory system	−0.522 ***
Role conflict→Collective efficacy	−0.354 ***
Cohesion→Collective efficacy	0.516 ***
Team conflict→Collective efficacy	−0.096

Note. ** *p* < 0.01, *** *p* < 0.001.

## Data Availability

The data presented in this study are available on request from the corresponding author.
